# Tissue microarray construction for salivary gland tumors study

**DOI:** 10.4317/medoral.18204

**Published:** 2012-08-28

**Authors:** Felipe Paiva-Fonseca, Oslei P. de-Almeida, Ana L C. Ayroza-Rangel, Pablo Agustin-Vargas

**Affiliations:** 1 DDS. Department of Oral Diagnosis, Oral Pathology Section, Piracicaba Dental School, State University of Campinas-São Paulo -Brazil; 2 DDS, PhD. Department of Oral Diagnosis, Oral Pathology Section, Piracicaba Dental School, State University of Campinas- São Paulo-Brazil; 3 DDS, PhD. Department of Pathology, State University of Western Paraná-Paraná-Brazil

## Abstract

Objective: To describe and discuss the design, building and usefulness of tissue microarray (TMA) blocks for the study of salivary gland tumors (SGTs). Study Design: Two hundred thirty-eight formalin-fixed, paraffin-embedded SGTs were arranged in blocks of TMA using a manual tissue arrayer. Three representative cores of 1.0, 2.0 or 3.0mm were taken from each original block and their characteristics were analyzed and described. Results: It was created 12 TMA blocks that presented highly representative neoplastic cylinders. However, those neoplasias rich in cystic spaces such as mucoepidermoid carcinoma and Warthin tumor presented more difficulties to be sampled, as the neoplastic tissue available was scarce. Tissue damage and loss during TMA construction was estimated as 3.7%. Conclusion: Representative areas of SGTs, with relatively small loss of tissue, can be obtained with the construction of TMA blocks for molecular studies. However, tumors rich in cystic spaces present more difficulties to be adequately sampled.

** Key words:**Tissue microarray, tma, salivary gland tumors, immunohistochemistry.

## Introduction

Salivary gland tumors (SGT) are uncommon neoplasms that account for 6% of all head and neck neoplasias and 0.3% of all cancers. The incidence of SGT per 100.000 persons is reportedly as 0.9 in women and 1.5 in men, with varied biological behavior and clinical outcome (1,2). The development and progression of SGTs, like other neoplasias result from multiple genetic alterations and molecular studies may help to understand the me-chanisms involved in their tumorigenesis.

The development of cDNA microarray and proteomic techniques, allowed the analysis of thousands of genes or proteins in one single experiment, facilitating the identification of molecules with potential clinical applications (3,4). In line with these new approaches, in 1998 Kononen et al. (5) promulgated the idea of translating the convenience of DNA microarrays to tissues. This new methodology allowed simultaneous screening of dozens of tumor specimens at once, being popularly called as tissue microarray (TMA) (6).

TMA technology has the potential to signiﬁcantly accelerate in situ studies of tissue specimens, to explore associations between molecular changes and clinic-pathological informations and to ensure preservation of unique and precious research materials. Briefly, tiny tissue cylinders are acquired from hundreds of different primary tumor blocks and arranged in a matrix conﬁguration within a recipient parafﬁn block. Sections from such TMA blocks can then be used for simultaneous in situ analyses of up to 1000 tissue specimens either at the DNA, RNA or protein levels; providing maximal use of limited tissue resources (3,6-8).

The use of this high-throughput technique significantly facilitates the identification of new molecular markers that could predict the clinical behavior of tumors, helping to better understand their pathogenesis and biological characteristics. However, very few studies have applied this technology in the evaluation of SGTs, and their known morphological heterogeneity could theoretically affect the validity of the results obtained. Therefore, the objective of this article is to present and discuss the design and building of TMA blocks of 238 SGTs, considering its relevant technical points.

## Material and Methods

From January 2001 to December 2011, 493 cases of SGTs were retrieved from the archives of the Oral Pathology Department of the Piracicaba Dental School (161 cases) and from a Surgical Pathology laboratory of the Brazilian Southern state of Paraná (332 cases). Histological preparations stained with H&E were reviewed by three oral pathologists and, when necessary, new cuts were performed and stained with periodic acid-Schiff and mucicarmine. All cases were classiﬁed according to the 2005 World Health Organization’s Histological Typing of Salivary Gland Tumors. Those primary SGTs that affected major or minor salivary glands were included in the study. Cases without enough tissue available for TMA construction, or those whose definitive salivary gland origin could not be confirmed, were excluded from the study. After this selection, 238 formalin-fixed, paraffin-embedded primary SGTs remained available for being arrayed.

Representative tumor areas were selected and marked on H&E-stained sections using an objective marker (1.8 mm; Nikon Corporation, Tokyo, Japan). The slide was then overlaid on the original paraffin block to determine the corresponding area to be used. TMA were constructed using a manual tissue arrayer (Sakura Co.; Japan). Three representative cylindrical cores of 1.0, 2.0 or 3.0 mm diameter were taken from each original tissue block and then arrayed sequentially into a recipient ready-to-use paraffin block (Sakura Co.; Japan). Two cores of normal parotid gland tissue and one of oral squamous cell carcinoma were inserted into the left upper corner of each recipient block as controls for future immunohistochemical reactions and for orientation when examining the slides. A map specifying the exact position of each case was made, to facilitate the interpretation of the histological and immunohistochemical results.

The current study has been approved by the Ethical Committee of the Piracicaba Dental School – State University of Campinas (Protocol 141/2011).

## Results

**Figure 1:**
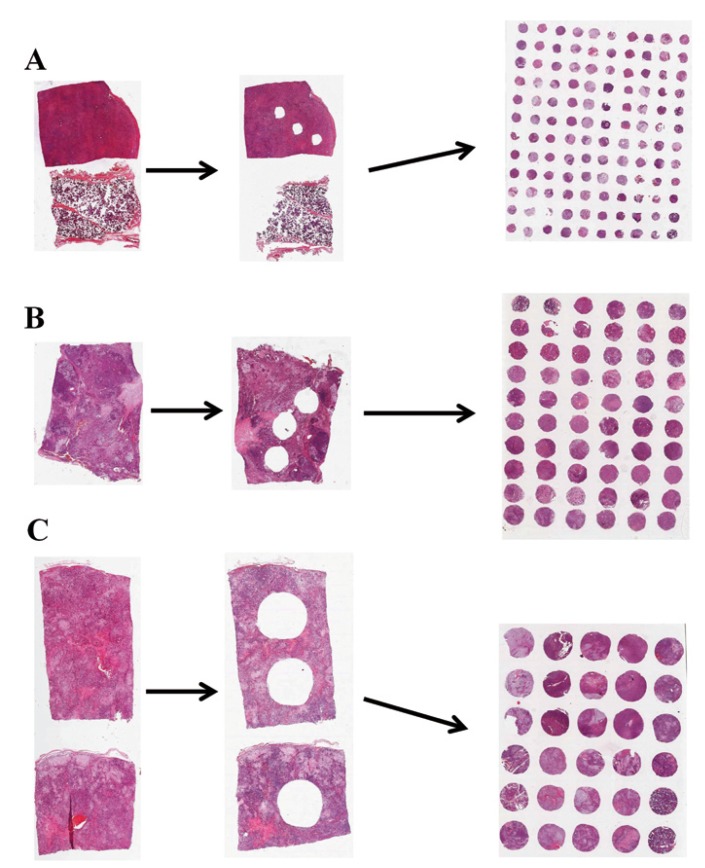
Example of full tissue sections previously and after the acquisition of TMA cores of A) 1.0, B) 2.0 and C) 3.0 mm. It can be seen that sections that provided larger cylinders became significantly more damaged than those that provided smaller cylinders. Microscopic images acquired using the Aperio ScanScope CS scanner.

Among the 238 SGTs used in the construction of the TMA blocks, there were 200 benign and 38 malignant tumors ( Table 1). 72.7% of the cases involved major salivary glands (173 cases), whereas 27.3% affected intra-oral minor glands (65 cases). It was built 12 TMA blocks; from these, it was created 8 blocks of pleomorphic adenoma (6 of 2.0mm cores, 1 of 1.0mm cores and 1 of 3.0mm cores), 1 block of Warthin tumor (2.0mm cores) and 1 block containing pleomorphic adenoma, Warthin tumor and canalicular adenoma (2.0mm cores). The last 2 blocks were composed by 2.0mm cores of malignant tumors that included mucoepidermoid carcinoma, adenoid cystic carcinoma, acinic cell carcinoma, polymorphous low-grade adenocarcinoma, epithelial-myoepithelial carcinoma, myoepithelial carcinoma, carcinoma ex-pleomorphic adenoma and adenocarcinoma not otherwise specified (Fig. 1).

**Table 1:**
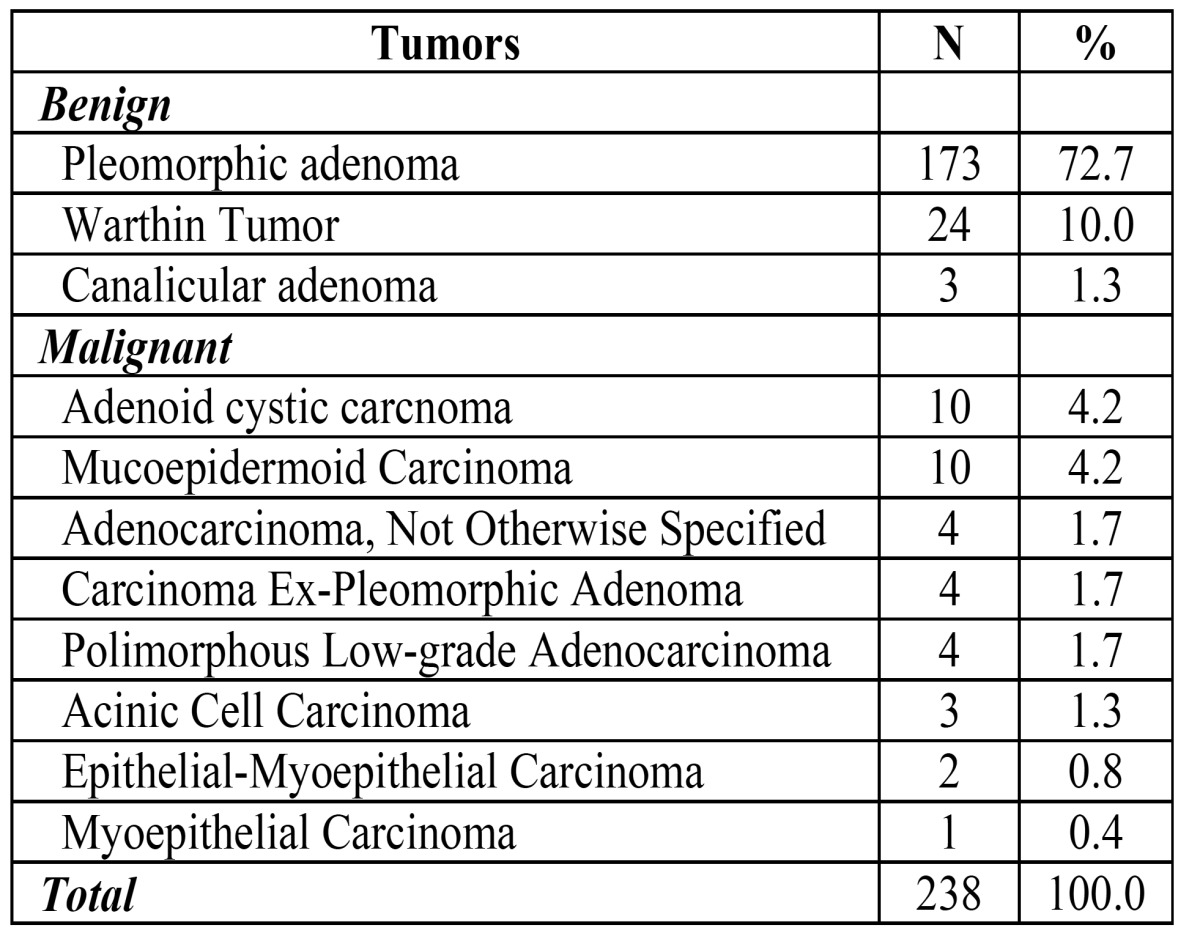
Histopathological distribution of the 238 salivary gland tumors used for construction of 12 TMA blocks.

Most of the time and efforts to construct the blocks were actually spent in the search, organization and pathological review of the tissue specimens to be included in the arrays, whereas the TMA building itself usually took from 1 to 2 hours for each block depending on the diameter of the cylinders used.

As expected, the use of larger needles caused more damage to the original tissue blocks. Hence, while those blocks that provided 1.0mm cores could be used in other projects, most of those specimens that provided 3.0mm cores could not be used again. In addition, larger needles substantially reduced the number of specimens that could be arrayed. TMA blocks composed by 2.0mm cylinders allowed as many as 60 specimens to be arrayed into a ready-to-use recipient block of approximately 45x20mm, what corresponded to 19 different cases plus controls. Those composed by 1.0mm cylinders allowed 120 specimens, representing 39 cases; whereas those TMAs composed by 3.0mm cylinders allowed only 30 specimens or 9 different cases, always using triplicate arrangement.

In approximately 5% of the cases there was a slight difference between the area selected in the H&E-stained slide to be inserted in the TMA block and the one that in fact was inserted. It was considered that the rate of tissue loss attributable to tissue damage during TMA construction was about 3.7%.

**Figure 2:**
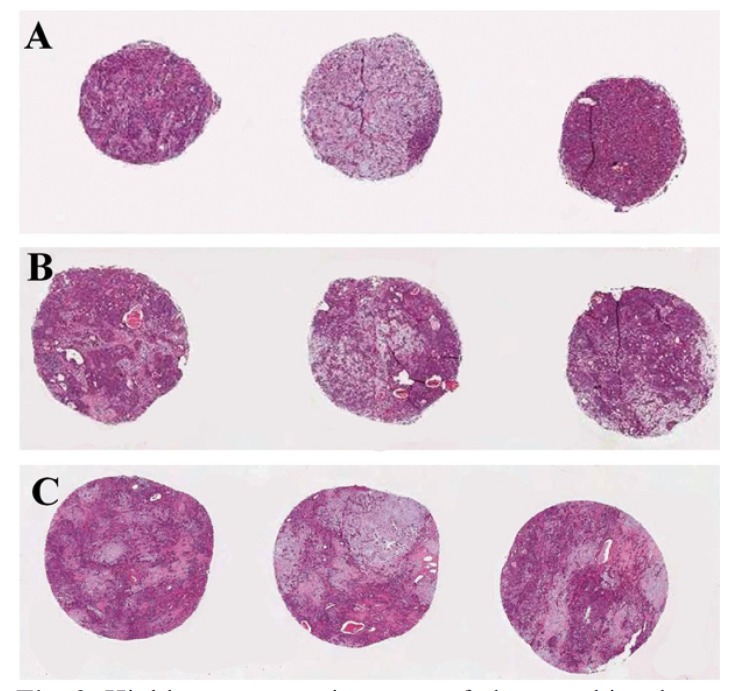
Highly representative areas of pleomorphic adenoma in tissue cores of 1.0, 2.0 and 3.0mm.

**Figure 3:**
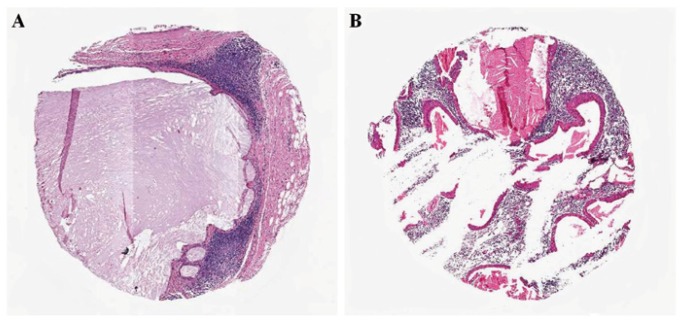
In cases rich in cystic spaces as low-grade mucoepidermoid carcinoma and Warthin tumor, only few neoplastic tissue are found on the TMA cores.

Most of the tumors were PA, and using three cores, highly representative areas of the tumor were obtained using either 1.0, 2.0 or 3.0 mm punches (Fig. 2). In tumors with a more homogenous morphological pattern, as canalicular adenoma and myoepithelial carcinoma, the representativity of the TMA was even higher. However, tumors rich in cystic spaces as low-grade mucoepidermoid carcinoma and Warthin tumor the tissue samples were not considered adequate, since only few neoplastic tissue was available in the TMA blocks (Fig.3).

## Discussion

Tissue microarrays (TMAs) were developed by Kononen et al. (5) and are now widely accepted as a fast and cost-effective tool that facilitates the analysis of molecular alterations in thousands of tissue specimens by acquiring cylindrical cores of formal infixed, paraffin-embedded tissue specimens and arraying them into a recipient block. These TMA blocks can be used for any in situ tissue analysis, including IHC, in situ hybridization, DNA ploidy, nuclear morphometry, and FISH (9).

During the last decade, numerous studies have validated this method in the investigative surgical pathology. TMA cores as small as 0.6mm have been confirmed to be adequate for analyzing breast cancer specimens by IHC for the expression of estrogen and progesterone receptors and the tyrosine kinase receptor HER-2 (10,11). Similarly, TMA immunohistochemical staining for p53, cyclin D1, bcl-2, bax, Cox-2, β-catenin, c-myc, PTEN and p-Akt1enabled high-throughput analysis of genetic alterations that might contribute to human colon cancer development and progression (12). TMA validation has also been conducted in endometrial cancer (13), esophageal squamous cell carcinoma (14), lung cancer (15), cervical adenocarcinoma (16), ovarian carcinoma (17) and in many other human neoplasias.

The use of TMA for molecular studies of SGTs is scarce ( Table 2) (1,18-25). Iwafuchi et al. (18) first used TMA for analyzing molecular features of these tumors. By evaluating the expression of a large panel of proteins in different salivary gland neoplasias, these authors concluded that SGTs may be well characterized using markers only toward myoepithelial, luminal and basal cells. Also by IHC and TMA, Mcm-2 has been proved to be a proliferative sensitive marker for SGTs and PLUNC proteins have been suggested to be useful diagnostic tools for mucoepidermoid carcinoma, whereas geminin has been strongly associated with reduced overall and relapse-free survival rates in patients affected by salivary gland carcinomas (1,19,20). Freier et al. (21) evaluated KIT expression in a large sample of histologically defined subgroups of adenoid cystic carcinoma, observing a stronger expression in cribriform and tubular subtypes when compared to the solid variant. Similarly, Freier et al. (22) analyzed the prevalence of chromosome 22q13 copy number gains in 70 ACC and found that it represents a decisive molecular event in early stages of ACC, irrespective of histologic differentiation. However, despite these interesting results previously described, there are no reports describing and evaluating the most important technical points to the construction of TMA blocks for studying SGTs.

**Table 2:**
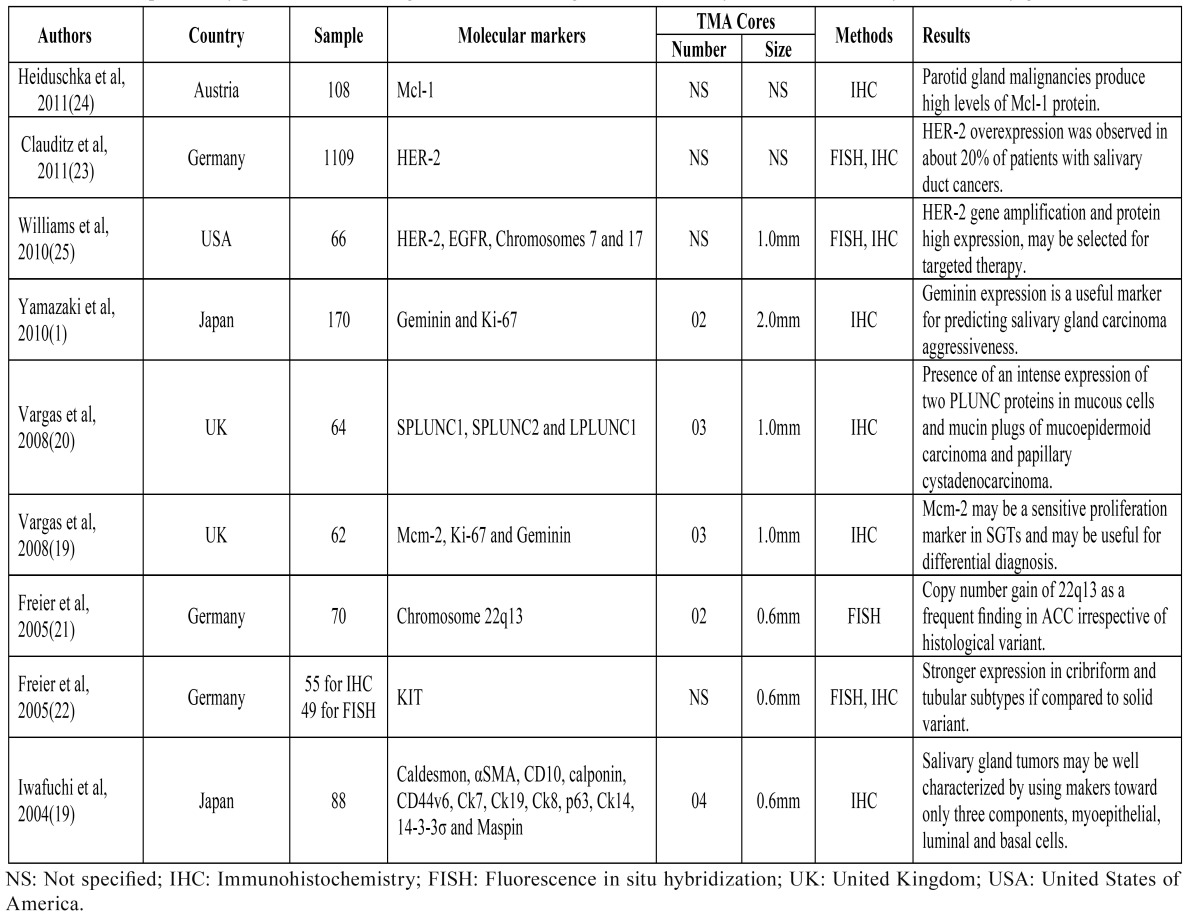
Studies previously published in the English literature using tissue microarray for molecular analysis of salivary gland tumors.

TMAs present various relevant advantages for molecular studies of paraffin embedded tissues. It permits the concomitant use of a large number of cases and significantly reduces the experimental handling time (3). Moreover, as the reactions are done in one single slide, the reagent concentrations, incubation times, temperature, wash conditions, and antigen retrieval are the same for all specimens. The necessary reagent volume is significantly reduced, making it a very cost-effective method (6,26). In our study, using ready-to-use recipient TMA blocks, it was possible to resume our whole 238 neoplastic samples to only 12 blocks, facilitating the evaluation of molecular markers in this large number of cases. In addition, the use of TMAs preserves precious and finite tissue resources and maximizes the number of experiments that can be performed with the material present in one paraffin block (6).

Whereas large tissue sections are used for histological diagnosis, TMA has been reserved for research purposes. However, tumor heterogeneity has traditionally been recognized as a potential problem for those using TMAs, and the most used 0.6mm cores have been perceived as too small and potentially not representative of the entire specimen. Taking multiple samples of each tumor seems to be the most direct way of combating the potential lack of representativeness in a certain tissue (7). Recent reports achieved 95% accuracy with only two cores, whereas most studies indicate that triplicate TMA cores have up to 98% concordance when compared with the results of full sections (3,16). Other alternative would be the use of larger punch needles; however, the increase in the number of cores and their diameters leads to a considerable damaged donor block and fewer samples arrayed (7). Evaluating the histological features present in the cores obtained in the present study, it could be noted that triplicate cores of 1.0, 2.0 and 3.0mm were all well representative of the original tissue, although 3.0mm cores evidently offered more neoplastic cells and structures to be evaluated than 1.0 and 2.0mm cores. However, only 9 cases could be inserted in a TMA recipient block when 3mm cylinders were used, whereas up to 39 could be arrayed when 1mm were taken; moreover, smaller diameters better preserved the donor blocks for future studies.

Improper selection of representative tumor areas on the H&E original slide by the pathologist, or incorrect punching of these representative areas can cause tissue cores that contain inadequate areas to be studied (6). In the current study, it was noted that in a small percentage of cases, the neoplastic area inserted in the recipient block was not the exact area selected in the H&E slide, what may also be attributable to differences in the tissue contraction in the original paraffin donor block.

Finally, due to the small size of the cylinders and the high number of samples, TMA cores are much more prone to be lost during sectioning than full sections. The total number of lost cores due to technical reasons has been estimated to vary from 4 to 23% (14,16,27). In the building process of the TMA blocks in the present study, 3.7% of the cores were lost; however, this rate would probably increase if the samples were submitted to IHC procedures.

In conclusion, tissue microarray is a high-throughput, cost-effective and tissue-saving technique in molecular analysis of forma-lin-fixed, paraffin-embedded neoplasias, helping to overcome the ordinary time-consuming work. The present study showed the usefulness of this technique in the construction of SGTs TMA blocks, revealing that solid tumors are more indicated to be mi-cro-arrayed than their cystic counterparts. These TMA blocks will now be used for immunohistochemical studies to better evaluate SGT’s molecular features.
